# Predictive Role of Haematological Determinants on Outcomes of Critically Ill COVID-19 Patients Admitted to Intensive Care Unit

**DOI:** 10.7759/cureus.16764

**Published:** 2021-07-30

**Authors:** Ahilanandan Dushianthan, Nabil Abdul, Josh Dmochowski, Izabela James, Lesley Heesom, Jennifer Westwood, Judith Effney, Sarah Bruty, Kordo Saeed, Savita Rangarajan, Rashid Kazmi

**Affiliations:** 1 Faculty of Medicine, University of Southampton, Southampton, GBR; 2 National Institute for Health Research Southampton Clinical Research Facility and National Institute for Health Research Southampton Biomedical Research Centre, University Hospital Southampton NHS Foundation Trust, Southampton, GBR; 3 General Intensive Care Unit, University Hospital Southampton NHS Foundation Trust, Southampton, GBR; 4 Haematology, University Hospital Southmapton, Southampton, GBR; 5 Haematology, University Hospital Southampton NHS Foundation Trust, Southampton, GBR; 6 Microbiology Innovation and Research Unit, University Hospital Southampton NHS Foundation Trust, Southampton, GBR

**Keywords:** covid 19, acute kidney injury care, d dimer, factor viii, acquired von willebrand, adamts13, intensive respiratory care

## Abstract

Background: The mortality of patients admitted to the intensive care unit (ICU) with COVID-19 remains significantly high. Severe COVID-19 pneumonia is characterised by refractory hypoxemia with significant shunting due to a combination of alveolar damage, vascular vasoconstriction, and occlusion due to microthrombi. Similar pathological features are seen in extra-pulmonary organs. However, the influence of thrombotic markers on the risk of mechanical ventilation (MV) and the development of acute kidney injury (AKI) is not fully defined.

Methods: This was a cross-sectional evaluation of haemostatic and thrombotic markers of COVID-19 patients admitted to the ICU to determine their predictability for the development of thromboembolism and the need for non-invasive or invasive MV, development of AKI, and mortality.

Results: An extended coagulation profile was obtained in 71 SARS-CoV-2 positive patients admitted to the ICU. All patients had acute severe hypoxic respiratory failure and required non-invasive or invasive MV. There were increases in peak D-dimer (3.0 mg/L), factor VIII levels (255 IU/dL) vWF antigen (471 IU/dL) with low ADAMTS13 activity (54.7 IU/dL) compared to the reference ranges. Peak D-dimer was consistently raised in patients who developed AKI and required invasive MV. ADAMTS13/vWF/platelet axis was associated with disease severity, multi-organ dysfunction, and mortality.

Conclusions: Haematological abnormalities are a common feature of severe COVID-19 pneumonia. We found peak D-dimer and vWF-ADAMTS13-platelet axis are associated with increased ICU severity and outcome in severe COVID-19 patients admitted to ICU. Larger studies are needed to evaluate this more comprehensively.

## Introduction

Despite advances in the knowledge and management of COVID-19 patients, the mortality among those admitted to the intensive care unit (ICU) continues to be significantly high [1]. The understanding of the COVID-19 pathophysiology has considerably evolved since its first clinical detection in Wuhan, and it is established that the primary target of the novel SARS-CoV-2 is the angiotensin-converting enzyme (ACE-II) receptor at nasal and alveolar epithelial cells [2]. However, the clinical manifestation is variable, with commonly mild or no symptoms to severe acute respiratory distress syndrome (ARDS). While only a few patients needing supplemental oxygen, even fewer require mechanical ventilatory support. As the disease progresses, the inflammatory response in the pulmonary bed intensifies and causes diffuse alveolar damage with hyaline membrane formation [3]. Additionally, capillary congestion with in situ micro-and macro-thrombi formation appears to be a significant contributory factor for organ damage [4,5]. These thrombogenic tendencies are not unique to pulmonary circulation. Indeed, extra-pulmonary thrombotic manifestations are also well documented in other organs involving the heart, kidneys, and brain, adding to the significant morbidity and mortality associated with COVID-19 critical illness [4,6,7].

Haematological abnormalities with an associated high incidence of thrombotic events are a well-recognised feature of SARS-CoV-2 infection [8]. However, the propensity for multi-organ failure in relation to coagulation variables is not fully explored, particularly in severe COVID-19 critical illness. Although this is likely a culmination of several pathological mechanisms, including a possible role of direct cell injury by SARS-CoV-2 virus causing multi-organ damage, autopsy findings have also suggested a prothrombotic coagulopathy due to microangiopathy in some patients [9].

While there is evidence showing that specific haemostatic markers to be associated with higher mortality and thromboembolism, their role in predicting the need for mechanical ventilation (MV) and development of acute kidney injury (AKI) is less clear. Consequently, in a cohort of COVID-19 patients admitted to ICU, we examined haematological markers that may predict these outcomes during their clinical course.

## Materials and methods

This is a retrospective study of adult COVID-19 (≥18 years) patients admitted to the General ICU at University Hospital Southampton between March 2020 and March 2021. The diagnosis was confirmed by nasopharyngeal swab specimens tested by real-time polymerase chain reaction (RT-PCR) for SARS-CoV-2. All patients had hypoxic respiratory failure and required either non-invasive or invasive MV. All clinical management was in accordance with local guidelines. In the absence of any contraindications, all patients had augmented thromboprophylaxis with either unfractionated heparin or low molecular weight heparin against anti-factor Xa target levels of 0.50-1.00 (IU/dL). This study was part of a larger observational cohort study (REACT-COVID Observational Database) and has the approval of the local research and development and National Health Research Authority (IRAS:285145) with a REC reference 20/HRA/2986. Due to the nature of the study, consent was waived. The reporting of this study adheres to the reporting of observational studies (Strengthening the Reporting of Observational Studies in Epidemiology, STROBE) guidelines.

A cross-sectional analysis of extended coagulation variables was performed as part of clinical management. These included international normalised ratio (INR), activated partial thromboplastin time ratio (APTR), fibrinogen, serial and admission platelets and D-dimer, factor VIII, vWF antigen, and ADAMTS13. The laboratory procedures for all haematological assays are detailed in Table 1. Patient demographics including age, gender, symptom onset, body mass index (BMI), ethnicity, and pre-existing comorbidities were collected. The comorbidities are numerically quantified for every individual patient using Charlson's Comorbidity Index (CCI) [10]. The severity of acute illness was presented as admission Acute Physiology and Chronic Health Evaluation-II (APACHE-II) and Sequential Organ Failure Assessment (SOFA) scores. The degree of hypoxia was assessed by the ratio of partial pressure of arterial oxygen to the fraction of inspired oxygen (PaO_2_/FiO_2_). Admission routine laboratory profiles are also collected. The reported outcomes are symptomatic thromboembolism, hospital mortality, need for invasive or non-invasive MV, development of AKI as defined by the Kidney Disease Improving Global Outcome (KDIGO) criteria [11], and the need for renal replacement therapy (RRT).

**Table 1:** Methodology for laboratory assays with analyser and reagents used. ADAMTS13: A disintegrin and metalloproteinase with a thrombospondin type 1 motif, member 13; APTR; activated partial thromboplastin time ratio; INR; international normalised ratio; vWF: von Willebrand Factor; PT: prothrombin time; APTT: automated activated partial thromboplastin time; CIA: chemiluminescent immunoassay.

Coagulation parameter	Analyser	Method	Reagents
INR	Instrumentation Laboratory (IL, Bedford, MA)* ACL TOP^®^	Automated PT photo-optical light absorbance	HemosIL^® ^RecombiPlasTin 2G
APTR	IL ACL TOP^®^	APTT photo-optical light absorbance	HemosIL^® ^SynthASil
D-dimer (mg/L)	IL ACL TOP^®^	Automated latex turbidimetric immunoassay	HemosIL^®^ D-Dimer HS
Fibrinogen (g/L)	IL ACL TOP	PT-derived fibrinogen photo-optical light absorbance	HemosIL^® ^RecombiPlasTin 2G
ADAMTS13 (IU/dL)	Werfen ACL AcuStar	Automated CIA	HemosIL^® ^AcuStar ADAMTS13 Activity kit
Antithrombin (IU/dL)	Werfen ACL TOP	Automated chromogenic	Instrumentation Laboratory HemosIL^® ^Liquid Antithrombin kit
Factor VIII assay (IU/dL)	IL ACL TOP	Automated one-stage factor assay based on modified APTT	HemosIL^® ^Factor VIII deficient plasma
Von Willebrand Factor (IU/dL)	IL ACL TOP	Automated latex immunoturbidimetric assay	HemosIL^® ^von Willebrand Factor Antigen Kit

Summary statistics are presented as median and interquartile ranges. Mann-Whitney U test was used to compare continuous variables between groups and Fisher's Exact test for categorical variables. Spearman's correlation coefficient r was used to assess the relationship between haematological variables. When there is the statistical significance of a variable between groups, the predictive value of haematological variables was determined by receiver operating characteristic (ROC) curves. Although the sample size calculations were limited by the patient availability, to demonstrate an AUC of 7.5 is significantly different from the null hypothesis value 0.5 (meaning no discriminating power) with an anticipated event rate of 20%, with α -0.05 and β-0.20 (power is 80%), the estimated sample size was 65. We categorised these values into binary models and used univariate logistic regression to assess the Odds ratio for individual outcomes. We did not perform multivariate regression analysis due to the small outcome sample size. All analyses were performed using GraphPad Prism (version 9.0.0), GraphPad Software, San Diego, and MedCalc (version 19.6.4, Statistical Software Ltd., Ostend, Belgium). A difference with a p-value of <0.05 was considered to be statistically significant.

## Results

There were 340 COVID-19 patients admitted to the ICU during the period between March 2020 and March 2021. However, a complete coagulation profile was only performed at the later stages of the pandemic, and as a result, only 71 patients were included in this study. All patients included in this study have completed their outcomes. The median age was 58 (IQR 53, 70) and 70% were male. The median duration of symptom onset prior to the ICU admission was seven days (IQR 6, 10). The majority were white Caucasians (77.5%) and 47.9% had a BMI of >30 kg/m^2^. The CCI, which is a cumulative weighted index used to predict the risk of one-year mortality depending on the comorbidity, was 2.0 (IQR 1.0, 3.0). The nadir PaO_2_/FiO_2_ ratio for the first 24 hours of admission to ICU was 9.8 (IQR 8.1, 13.6) kPa. The detailed demographics, admission baseline characteristics, and routine laboratory variables of all included patients are summarised in Table 2.

**Table 2:** Patients' demographics and baseline clinical and laboratory variables. Data are presented as median and interquartile ranges. The acute severity indicators APACHE II, SOFA score, and PaO_2_/FiO_2_ ratio are based on the data from the first 24 hours of intensive care. APACHE II: Acute Physiology and Chronic Health Evaluation II; BMI: body mass index; LDH: lactate dehydrogenase; PaO_2_/FiO_2_: partial pressure of arterial oxygen to fraction of inspired oxygen; SOFA: Sequential Organ Failure Assessment Score; CCI: Charlson’s comorbidity index.

Demographics	All patients (N=71)
Age (years)	58 (53, 70)
Male (%)	50 (70%)
Symptomatic days prior to hospitalisation	7 (6,10)
BMI >30 kg/m^2^, n (%)	34 (47.9%)
White Black Asian; Indian; other; unknown	55 (77.5%); 3 (4.2%); 11 (15.5%); 2 (2.8%)
CCI	2 (1.0, 3.0)
Admission APACHE II score; admission SOFA score; admission nadir PaO_2_/FiO_2_ ratio (kPa)	13 (9, 18); 5 (3, 7); 9.8 (8.1, 13.6)
Bilirubin (µmol/l)	10 (8, 14.8)
Creatinine (µmol/l)	74 (55, 95)
Creatinine kinase (U/l)	216 (93, 380)
C-reactive protein (mg/l)	136 (90, 212)
Ferritin (mg/l)	791 (456, 1348)
HbA1c (mmol/mol)	47.0 (41, 55)
LDH (U/l)	1025 (804, 1331)
Lymphocytes × 10^9^/l	0.7 (0.6, 1.0)
Neutrophil/lymphocyte ratio	9.1 (5.9, 15.2)
Procalcitonin (ng/ml)	0.6 (0.2, 1.5)
Troponin (ng/l)	16.0 (8, 39)
White cell counts × 10^9^/l	8.5 (6.0, 11.7)

Of those who had augmented coagulation profile screened, 17 patients (23.9%) were diagnosed with a clinically symptomatic thromboembolic process during their admission to the ICU. Patients were not routinely screened for venous or arterial thromboembolism and only had subsequent investigations following a positive symptom or sign. All patients had augmented prophylactic anticoagulation either in the form of low molecular weight heparin (enoxaparin) or unfractionated heparin. Eight patients had symptomatic deep venous thrombosis and five had a pulmonary embolism. One patient had both DVT and PE. Three patients had acute strokes and one of those had an additional diagnosis of deep venous thrombosis of the leg. All patients had acute respiratory failure and needed respiratory support either in the form of non-invasive ventilation (15.5%) or invasive MV (84.5%). Forty-six patients (64.8%) developed AKI as defined by the 2012 Kidney Disease Improving Global Outcomes (KDIGO) criteria at any point during their ICU admission and 13 of those (18.3%) needed RRT. Of those patients who had augmented coagulation screening, 74.6% survived hospital discharge. All ICU outcomes are summarised in Table 3.

**Table 3:** Overall clinical outcomes. MV: mechanical ventilation; AKI: acute kidney injury; RRT: renal replacement therapy.

Outcomes	Number of patients, N=71
Thromboembolism	17 (23.9%)
Non-invasive mechanical ventilation only	11 (15.5%)
Invasive MV	60 (84.5%)
AKI at any time during admission, any stages	46 (64.8%)
RRT	13 (18.3%)
ICU survival	55 (77.5%)
Hospital survival	53 (74.6%)

**Figure 1:**
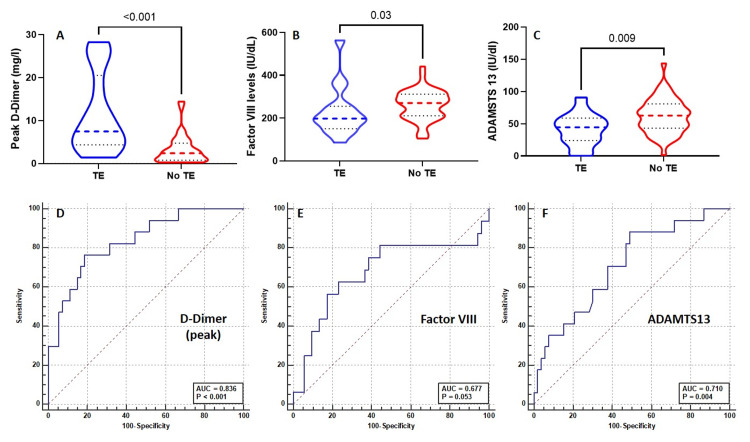
The differences in the peak D-dimer levels (A), factor VIII (B), and ADAMTS13 activity (C) between patients with and without symptomatic thromboembolism and the ROC curves for peak D-dimer (D), Factor VIII (E), and ADAMTS13 (E) for the prediction of thromboembolism. The comparison was made by the Mann-Whitney U test. ADAMTS13: A disintegrin and metalloproteinase with a thrombospondin type 1 motif, member 13; vWF: von Willebrand Factor; TE: thromboembolism.

Detailed median laboratory measurements in comparison to the established reference ranges are presented in Table 4. The median time from hospitalisation to laboratory analysis of vWF, ADAMTS13, and FVIII was seven days. The rest of the coagulation markers were performed on a daily basis until ICU discharge. The median time frame from hospital admission to the peak D-dimer levels and nadir platelet counts were six days and three days, respectively. Patients with symptomatic thromboembolism (TE) had elevated peak D-dimer levels of 7.5 mg/L (IQR 5.4, 17.7) compared to those without (2.4 mg/L, IQR 0.8, 4.6; p<0.001; Figure 1). Moreover, factor VIII levels were much lower in patients with TE (198 IU/dL vs 270 IU/dL, p=0.03). Patients with TE had lower ADAMTS13 (44.5 IU/dL vs 62.7 IU/dL, p=0.009) and a trend towards higher vWF/ADAMTS13 ratios in patients with thromboembolism (12.0 vs 5.8, p=0.05), and was statistically not significant (Figure 1). ROC was conducted for the peak D-dimer levels, ADAMTS13, and factor VIII levels for the development of thromboembolism, which revealed D-dimer as the best predictor with an area under the curve (AUC) of 0.836 (95% CI 0.729-0.913, p<0.0001). The cut-off value for D-dimer of ≥5.2 mg/L has a sensitivity of 76% and specificity of 82% for the development of thromboembolism (Figure 1). This was followed by ADAMTS13 with an AUC of 0.710 (95% CI 0.568-0.851, p=0.004) at a level of ≤62 IU/dL and factor VIII levels of ≤210 IU/dL (with an AUC of 0.677 (95% CI 0.498-0.856, p=0.053). Using these cut-off levels, univariate logistic regression analysis for these variables suggests a peak D-dimer of ≥5.2 mg/L (odds ratio: 14.3, 95% CI 3.84-53.23, p<0.001), ADAMTS13 ≤62 IU/dL (odds ratio: 5.23, 95% CI 1.34-20.34, p=0.008), and factor VIII levels of ≤210 IU/dL (odds ratio: 3.96, 95% CI 1.23-12.74, p=0.02) were associated with increased risk of thromboembolism.

**Table 4:** Coagulation profile between patients who developed symptomatic thromboembolism and patients without thromboembolism. *Assessed by the Mann-Whitney U test. ADAMTS13: A disintegrin and metalloproteinase with a thrombospondin type 1 motif, member 13; APTR: activated partial thromboplastin time ratio; INR: international normalised ratio; vWF: von Willebrand Factor.

Coagulation profile	Patients with thromboembolism (N=17)	Patients without thromboembolism (N=54)	p-Value*
D-dimer (admission) mg/L	1.1 (0.5, 4.0)	0.6 (0.3, 1.0)	0.02*
D-dimer (peak) mg/L	7.5 (5.4, 17.7)	2.4 (0.8, 4.6)	<0.001*
Platelets (admission) 10^9^/L	202 (170, 263)	191 (148, 262)	0.92
Platelets (nadir) 10^9^/L	157 (100, 197)	151 (112, 190)	0.84
Fibrinogen g/L	7.6 (7.0, 7.9)	7.9 (6.4, 8.0)	0.81
ADAMTS13 IU/dL	44.5 (25.7, 57.1)	62.7 (43.4, 80.4)	0.009*
Factor VIII assay IU/dL	198.0 (167.5, 248.5)	270.5 (211.0, 310.8)	0.03*
vWF antigen IU/dL	548.0 (307.2, 674.0)	469.0 (221.0, 606.0)	0.86
vWF/ADAMTS13	12.0 (6.0, 18.9)	5.8 (4.5, 11.8)	0.05

**Figure 2:**
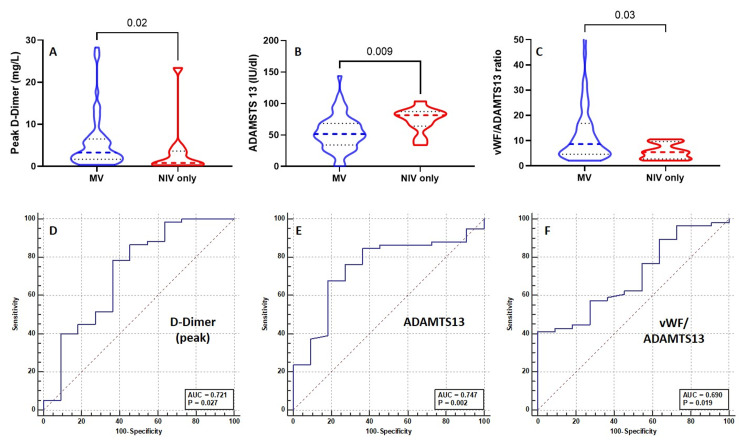
The differences in the peak D-dimer levels (A), ADAMTS13 activity (B), and vWF/ADAMTS13 ratio (C) between patients with and without the need for invasive mechanical ventilation and the ROC curve for peak D-Dimer (D), ADAMTS13 activity (E), and vWF/ADAMTS13 ratio (F) for the prediction of invasive mechanical ventilation. The comparison was made by the Mann-Whitney U test. ADAMTS13: A disintegrin and metalloproteinase with a thrombospondin type 1 motif, member 13; vWF: von Willebrand Factor; MV: invasive mechanical ventilation; ROC: receiver operating characteristic.

Patient with severe hypoxic respiratory failure is likely to require non-invasive or invasive mechanical ventilation to provide adequate oxygenation. Indeed, patients who require MV are more likely to be critically ill with more adverse outcomes. All patients received NIV on admission and patients who deteriorated while receiving NIV were subsequently endotracheally intubated and MV. We compared the laboratory variables between those who had NIV alone with patients requiring eventual invasive MV. Once again, the peak D-dimer levels were much higher for patients requiring MV than NIV (3.3 mg/L vs 0.80 mg/L, p=0.02). The ADAMTS13 levels were much lower (51.6 IU/dL vs 81.4 IU/dL, p=0.009) with a higher vWF/ADAMTS13 ratio (8.9 vs 5.4, p=0.03) in MV-treated patients. (Figure 2). The AUC for the risk of invasive MV for ADAMTS13 levels was 0.747 (95% CI 0.592-0.901, p=0.002) at a cut-off level of ≤63 IU/dL with a sensitivity and specificity of 68% and 81%, respectively (Figure 2). For the outcome risk of MV, the AUC for peak D-dimer and vWF/ADAMTS13 ratio was 0.721 (95% CI 0.526-0.917, p=0.027, cut-off: ≥1.6 mg/L) and 0.690 (95% CI 0.540-0.851, p=0.019, cut-off: ≥10.5), respectively (Figure 2). Univariate logistic regression analysis showed an increased risk of MV in ADAMSTS13 levels of ≤63 IU/dL (odds ratio: 7.57, 95% CI 1.50-38.26, p=0.005), and vWF/ADAMTS1 ratio of ≥10.54 (odds ratio: 6.97, 95% CI 0.833-58.27, p=0.027) were associated with increased risk of MV. 

**Figure 3:**
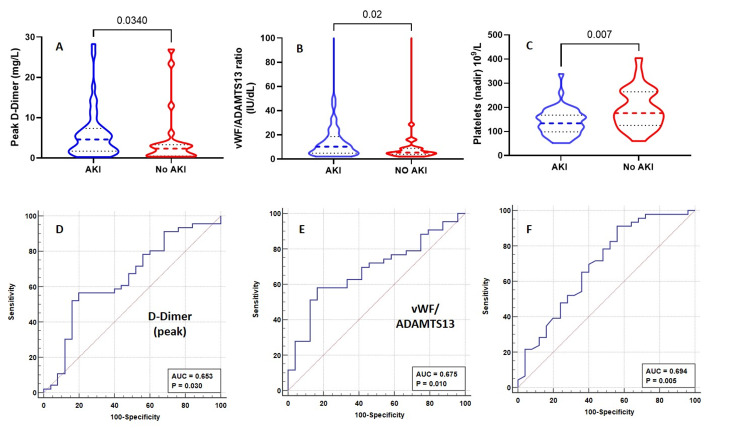
The differences in the peak D-dimer levels (A), vWF/ADAMTS13 ratio (B), and nadir platelets (C) between patients with and without acute kidney injury and the ROC curves for peak D-dimer (D), vWF/ADAMTS13 ratio (E), and nadir platelets (F) for the development of acute kidney injury. The comparison was made by the Mann-Whitney U test. ADAMTS13: A disintegrin and metalloproteinase with a thrombospondin type 1 motif, member 13; vWF: von Willebrand Factor; ROC: receiver operating characteristic.

Among COVID-19 critically ill patients, the development of AKI is associated with a significant increase in mortality. Among those patients screened, 64.8% developed an AKI at any time during their ICU admission and 18.3% needed RRT (Table 3). The development of AKI at any time during the admission was associated with an elevated peak D-dimer (4.6 mg/L vs 2.3 mg/L, p=0.03), thrombocytopenia (platelets nadir: 134 × 109/L vs 176 × 109/L, p=0.007) vWF/ADAMTS13 ratio (10.3 vs 5.3, p=0.02) and a trend towards lower ADAMTS13 activity (51.6 IU/dL vs 66.1 IU/dL, p=0.13; Figure 3 and Tables 4-7). The ROC analysis for the peak D-dimer levels (AUC 0.653, 95% CI 0.515-791, P=0.03, cut-off ≥3.3 mg/L), nadir platelets (AUC 0.694, 95% CI 0.559-0.828, p=0.005, cut-off ≤196 × 109/L), and vWF/ADAMTS13 ratio (AUC 0.675, 95% CI 0.552-0.812, P=0.010 cut-off of ≥9) is presented in Figure 3. Univariate logistic regression analysis showed lower nadir platelets of ≤196 × 109/L (odds ratio: 5.24, 95% CI 1.63-16.81, p=0.004), and vWF/ADAMTS1 ratio of ≥9 (odds ratio: 6.32, 95% CI 1.845-21.62, p=0.0013) and D-dimer of ≥3.3 mg/L (odds ratio: 2.76, 95% CI 0.993-7.68, p=0.046) were associated with increased risk of AKI.

**Table 5:** Coagulation profile between patients who had mechanical ventilation and non-invasive ventilation. *Assessed by the Mann-Whitney U test. ADAMTS13: A disintegrin and metalloproteinase with a thrombospondin type 1 motif, member 13; APTR: activated partial thromboplastin time ratio; INR: international normalised ratio; vWF: von Willebrand Factor.

Coagulation profile	MV (N=60)	NIV alone (N=11)	p-Value*
D-dimer (admission) mg/L	0.7 (0.4, 1.9)	0.4 (0.3, 1.0)	0.26
D-dimer (peak) mg/L	3.3 (1.7, 6.3)	0.8 (0.4, 3.3)	0.02*
Platelets (admission) 10^9^/L	196 (162, 259)	169 (124, 315)	0.77
Platelets (nadir) 10^9^/L	153 (112, 189)	131 (104, 229)	0.81
Fibrinogen g/L	7.8 (7.0, 8.0)	7.9 (7.5, 8.0)	0.93
ADAMTS13 IU/dL	51.6 (35.1, 68.4)	81.4 (66.4, 85.8)	0.009*
Factor VIII assay IU/dL	256.0 (198.0, 308.0)	221.0 (203.5, 316.0)	0.84
vWF IU/dL	508.0 (318.7, 620.0)	384.0 (214.5, 485.5)	0.15
vWF/ADAMTS13	8.9 (4.7, 17.1)	5.4 (2.9, 7.8)	0.03*

**Table 6:** Coagulation profile between patients who developed acute kidney injury and patients without acute kidney injury. *Assessed by the Mann-Whitney U test. ADAMTS13: A disintegrin and metalloproteinase with a thrombospondin type 1 motif, member 13; APTR: activated partial thromboplastin time ratio; INR: international normalised ratio; vWF: von Willebrand Factor.

Coagulation profile	Patients with AKI (N=46)	Patients without AKI (N=25)	p-Value*
D-dimer (admission) mg/L	0.6 (0.3, 1.4)	0.6 (0.3, 2.5)	0.81
D-dimer (peak) mg/L	4.6 (1.7, 7.2)	2.3 (0.6, 2.5)	0.03*
Platelets (admission) 10^9^/L	186 (148, 242)	246 (170, 278)	0.05
Platelets (nadir) 10^9^/L	134 (99, 167)	176 (131, 263)	0.007*
Fibrinogen g/L	7.9 (7.0, 8.0)	7.4 (6.5, 8.0)	0.37
ADAMTS 13 IU/dL	51.6 (34.1, 68.6)	66.1 (49.3, 80.4)	0.13
Factor VIII assay IU/dL	259.0 (205.5, 311.5)	254.0 (196.0, 290.0)	0.65
vWF IU/dL	554.2 (371.6, 674.0)	430.4 (214.0, 482.0)	0.11
vWF/ADAMTS13	10.3 (4.9, 18.1)	5.4 (4.3, 8.6)	0.02*

**Table 7:** Coagulation profile between patients who died and survived. *Assessed by the Mann-Whitney U test. ADAMTS13: A disintegrin and metalloproteinase with a thrombospondin type 1 motif, member 13; APTR: activated partial thromboplastin time ratio; INR: international normalised ratio; vWF: von Willebrand Factor.

Coagulation profile	Alive (N=54)	Dead (N=11)	p-Value*
D-dimer (admission) mg/L	0.6 (0.3, 1.4)	0.6 (0.3, 1.9)	0.77
D-dimer (peak) mg/L	2.6 (0.8, 5.1)	5.5 (3.0, 6.5)	0.02*
Platelets (admission) 10^9^/L	214 (169, 267)	145 (127, 199)	0.006*
Platelets (nadir) 10^9^/L	165 (129, 197)	95 (80, 121)	<0.001*
Fibrinogen g/L	7.8 (7.0, 8.0)	7.6 (6.5, 8.0)	0.83
ADAMTS13 IU/dL	54.6 (43.3, 73.7)	50.6 (27.7, 72.4)	0.47
Factor VIII assay IU/dL	254.0 (208.5.0, 302.5)	259.0 (203.5, 316.0)	0.73
vWF IU/dL	459.6 (217.2, 578.4)	541.1 (377.7, 700.6)	0.15
vWF/ADAMTS13	6.0 (4.4, 15.0)	10.1 (5.6, 21.0)	0.21

**Figure 4:**
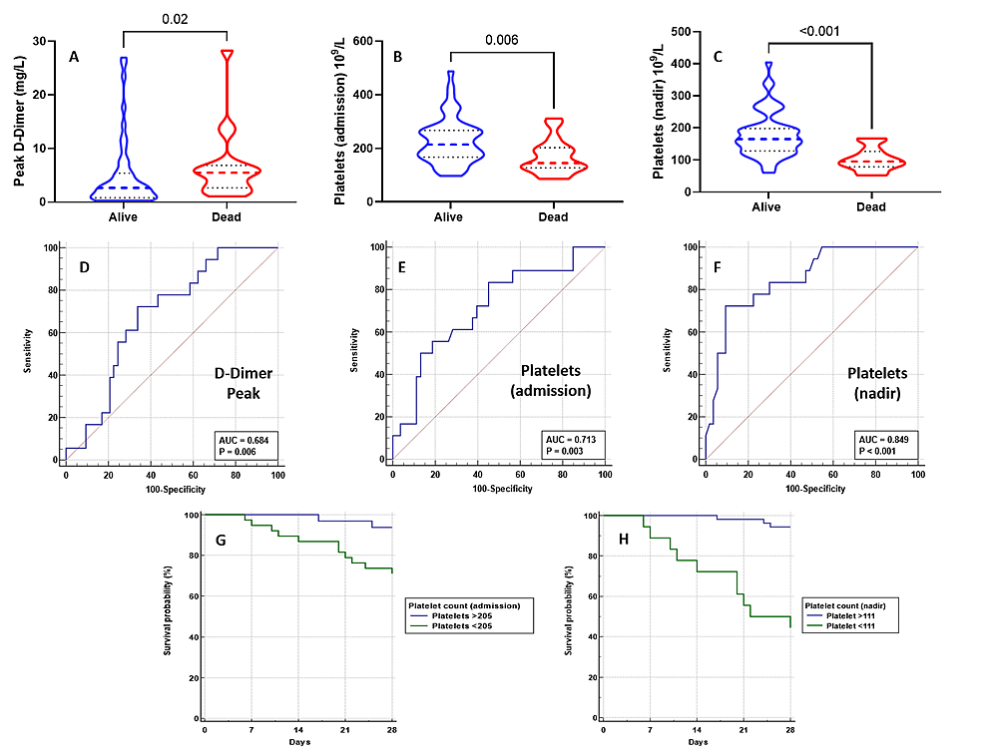
Levels of peak D-dimer (A), admission (B), and nadir platelets (C) and ROC analysis for peak D-dimer (D), admission platelets (E), and nadir platelets (F) between survivors and non-survivors. The Kaplan-Meier survival analysis according to the cut-off values for admission and nadir platelet values generated during ROC analysis (G, H, respectively). The comparison was made by the Mann-Whitney U test. ADAMTS13: A disintegrin and metalloproteinase with a thrombospondin type 1 motif, member 13; vWF: von Willebrand Factor.

The overall ICU and hospital survival rates for these critically ill COVID-19 patients were 77.5% and 74.6%, respectively. The haematological variables with significant difference between non-survivors and survivors were the peak D-dimer (5.5 mg/L vs 2.6 mg/L, p=0.02), admission platelets (145 × 109/L vs 214 × 109/L, p=0.006), and nadir platelets (95 × 109/L vs 165 × 109/L, p<0.001). Although there was a trend towards increased vWF/ADAMTS13 ratio among the non-survivors (10.1 vs 6.0, p=0.21), this was not significant. There was no difference in the ADAMTS13 activity between survivors and non-survivors. The ROC analysis for peak D-dimer levels (AUC 0.684, 95% CI 0.554-815, P=0.006, cut-off ≥3.3mg/L), admission platelets (AUC 0.713, 95% CI 0.572-0.854, p=0.003, cut-off ≤205 × 109/L), and nadir platelets (AUC 0.849, 95% CI 0.749-0.949, P<0.001 cut-off of ≤111 × 109/l) are presented in Figure 4. Using these cut-off values, univariate logistic regression analysis showed an increased risk of mortality with lower nadir platelets of ≤111 × 109/L (odds ratio: 24.96, 95% CI 6.26-99.49, p<0.0001) and admission platelets of ≤205 × 109/L (odds ratio: 6.30, 95% CI 1.63-24.44, p=0.0028). The peak D-dimer value (≥3.3 mg/L) was not significant (odds ratio: 2.82, 95% CI 0.92-8.65, p=0.06). The Kaplan-Meier survival analysis according to the admission platelet levels (205 × 109/L) and nadir platelets (111 × 109/L) is shown in Figure 4. A detailed summary of the coagulation profile for patients with thromboembolism, MV, AKI, and hospital mortality are presented in Tables 4-7.

**Figure 5:**
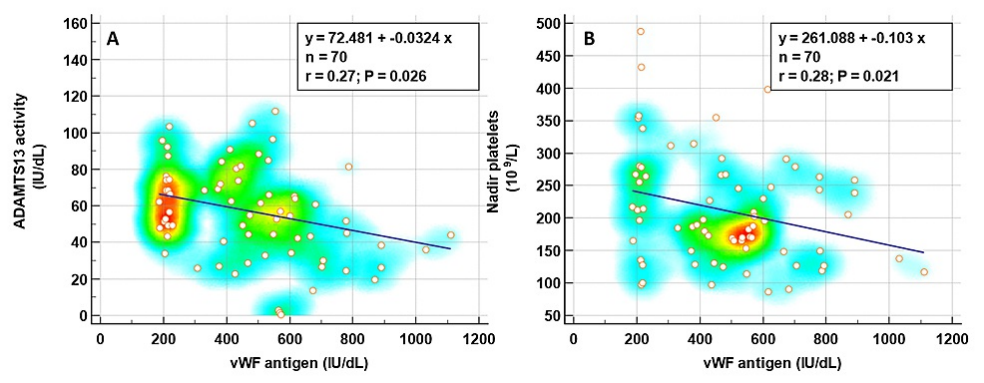
The correlation of vWF with ADAMTS13 and nadir platelet counts.

We further explored the relationship between the vWF antigen with ADAMTS13 activity and nadir platelet counts by using Pearson's correlation coefficient. There was an inverse relationship between vWF antigen and ADAMTS13 activity with a Pearson correlation r of −0.27, p=0.026. A similar inverse association was also seen with vWF and nadir platelet count with a Pearson r of 0.28 and p=0.021 (Figure 5).

## Discussion

In this small, single-centre, cross-sectional study of critically ill COVID-19 patients, we have found significant alterations in coagulation profiles with associated indices of increased severity of illness and clinical outcomes. Compared to the locally derived and manufacturers ranges, we found elevated levels of factor VIII and vWF antigen with lower ADAMTS13 activity in this group. Both admission and peak D-dimer levels are elevated from standard ranges. Patients with symptomatic thromboembolism had higher peak D-dimer levels and lower factor VIII and ADAMTS13 activity. Moreover, invasive MV and the development of AKI were associated with elevated peak D-dimer levels. A peak D-dimer value of ≥5.2 mg/L, ≥1.6 mg/L, and ≥3.3 mg/L had predictive probability for the development of symptomatic thromboembolism and organ dysfunction requiring MV and AKI development, respectively. Lower levels of ADAMTS13 activity were associated with invasive MV compared to patients who received NIV only. In these critically ill patients with severe hypoxic respiratory failure, ADAMTS13 activity of ≤63 IU/dL increased the risk of invasive MV with an odds ratio of 7.57. Moreover, the increased vWF/ADAMTS13 ratio was predictive of both the need for MV and AKI development. Overall, peak D-dimer levels and the vWF/ADAMTS13 ratio were associated with the severity of critical illness in COVID-19 patients with hypoxic respiratory failure in an ICU setting. However, hospital mortality was only associated with lower nadir platelet levels (≤111 × 109/L), admission platelets (≤205 × 109/L), and peak D-dimers. Univariate unadjusted analysis showed that an admission platelet count of <205 × 109/L and nadir platelet levels of ≤111 × 109/L were associated with a significant increase of hospital death with an odds ratio of 6.30 and 24.96, respectively.

While the understanding of the pathophysiology of COVID-19 continues to evolve, several laboratory markers of disseminated intravascular coagulation (DIC) have been shown to predict the prognosis adversely. Similar to our findings, one of the earliest studies showed that D-dimer levels over 1 mg/L at admission predicted an 18-fold increase in odds of dying before discharge among 191 COVID-19 patients seen at two hospitals in Wuhan, China [12]. In addition to a theme of coagulopathy with DIC underpinning the case fatality, various thrombotic events including pulmonary embolism, microvascular thrombosis and occlusion, central line thrombosis, and ischaemic strokes have been observed in these cases [13]. Moreover, antiphospholipid antibodies have also been reported in COVID-19 patients with multiple cerebral and cerebellar infarcts and may indicate another emerging pathophysiologic mechanism contributing to thrombotic tendency [14].

The integrity of haemostatic balance has been evaluated by assessing pro-haemostatic and anti-haemostatic markers. High factor VIII, von Willebrand factor levels, and low protein S levels have been consistently reported in patients with COVID-19, but their role in the pathogenesis is unclear [15,16]. Similar to our study, inverse correlation with von Willebrand cleaving protease, ADAMTS13, and vWF antigen has consistently been associated in both hospitalised and intensive care COVID-19 patients [17-20]. This, together with high vWF levels, suggests a possible role in the imbalance in the haemostatic axis, causing a micro and macrovascular prothrombotic state at an alveolar level predisposing to a severe hypoxic state.

In human umbilical vein endothelial cells (HUVEC) under static conditions, ADAMTS13 is constitutively produced at a slow rate and is not stored [21]. Conversely, vWF is produced and then stored in Wiebel Palade bodies. Under normal conditions, the balance between vWF and ADAMTS13 is such that there is no accumulation of ultra-large multimers of vWF. However, upon stimulation, the constitutive ADAMTS13 production appears to be overwhelmed by a marked release of vWF, by virtue of vWF storage without increases in ADAMTS13 [21]. Perhaps this explains a pathophysiological mechanism in conditions like COVID-19, particularly within the organ microvasculature where platelet-vWF plug formation prevents the systemic pool of ADMATS13 produced predominantly by hepatic stellate cells (HSC) from reaching distal areas, which further compounds thrombosis [21]. Furthermore, incubation of HSCs and HUVEC cells with inflammatory cytokines such as interferon (IFN)-gamma, tumour necrosis factor (TNF)- alpha, and interleukin-4 (IL4) also appear to reduce ADAMTS13 mRNA as detected by RT-PCR [22]. Whether the vWF/ADAMTS13 ratio varies within different areas of the vasculature, being dependent upon the effects of different combinations of cytokines and different vessel sizes, requires further research. However, this notion perhaps explains why the vWF/ADAMTS13 ratio was significant in patients requiring MV and RRT, where disruption to the integrity of blood flow in the microvasculature through microthrombosis leads to organ failure.

Regarding non-survivors, the relationship with lower admission platelet counts and a greater drop in platelet count was statistically significant. Given the excess vWF because of failure to increase the amount of ADAMTS13 proportionately, it is not unreasonable to assume that the former is responsible for platelet consumption. Indeed, a low admission platelet count and greater platelet nadir in non-survivors compared to survivors was clinically significant. Furthermore, there was also a clinically significant correlation between platelets and vWF in this study, perhaps making a vWF/platelet ratio a helpful predictor of mortality.

We examined the levels of platelets, D-dimers, FVIII, vWF antigen, and ADAMTS13 in critically ill patients with established COVID-19 admitted to ICU. Our results confirmed the previously reported findings of the derangement in these parameters [15-19]. However, we also analysed our data to determine a predictive value of the coagulation factor parameters for the occurrence of thromboembolism, MV requirement, and development of AKI. Our results show that the development of AKI and the requirement for MV was associated with high D-dimers and vWF/ADAMTS13 ratio. This finding is similar to previously published data of COVID-19 hospitalised patients and the risk of progression into AKI when there is a relative deficiency of ADAMTS13 activity [23]. This is an intriguing finding and indicates a possible therapeutic role for recombinant ADAMTS13 in preventing AKI and the need for invasive MV. It is worth noting that thromboembolism and AKI developed in patients despite augmented thromboprophylaxis, which supports exploring alternative pathways to prevent prothrombotic complications.

The correlation between vWF and platelets and the vWF/platelet ratio might also suggest that targeting this axis with caplacizimab is now used in the treatment of thrombotic thrombocytopenic purpura (TTP) may be of benefit [24]. This humanised bivalent variable-domain containing immunoglobulin fragment binds to the A1 domain of vWF and prevents its interaction with the GPIb-IX-V receptor on platelets, thereby preventing platelet activation and thrombosis [25]. The use of this nanobody could also circumvent the issues around ADAMTS13 inhibition by inflammatory cytokines such as IL-6 and by-products of NETosis (neutrophil extracellular trap) such as hypochlorous acid capable of oxidising methionine residues at positions 249, 331, and 496 to which recombinant ADAMTS13 might also be susceptible and therefore rendered suboptimal [26,27].

There were several limitations to our study. First, this was a small cross-sectional study, and second, due to the pragmatic observational nature, we were not able to screen all patients admitted to ICU and standardise the timeline for the detailed haematological analyses between patients. However, the median time from hospitalisation to laboratory analysis of vWF, ADAMTS13, and FVIII was seven days. Platelet count, D-dimer levels were performed on a daily basis until ICU discharge. Third, we did not perform longitudinal screening, which could have informed the dynamic fluctuations with clinical correlates during recovery. Fourth, we were not able to perform multivariate models adjusting for covariates due to the small sample size. Nevertheless, our findings are consistent with previously published data and we feel that the correlation of coagulation variables to the disease severity and mortality cannot be dismissed and should be the subject of larger studies.

## Conclusions

Although the primary target of SARS-CoV-2 being the lung, the progressive disease results in a proportion of patients developing multi-organ failure. The clinical course of these patients is variable, and several markers have been postulated to predict the outcome. Many haematological abnormalities have been observed in this cohort; some are of unknown significance, whereas others correlate with the severity and outcome. We found vWF-ADAMTS13-platelet axis is associated with increased ICU severity and outcome in severe COVID-19 patients admitted to ICU. Larger studies are needed to evaluate this more comprehensively.

## References

[1] (2021). COVID-19 report. https://www.icnarc.org/our-audit/audits/cmp/reports.

[2] Hamming I, Timens W, Bulthuis ML, Lely AT, Navis G, van Goor H (2004). Tissue distribution of ACE2 protein, the functional receptor for SARS coronavirus. A first step in understanding SARS pathogenesis. J Pathol.

[3] Carsana L, Sonzogni A, Nasr A (2020). Pulmonary post-mortem findings in a series of COVID-19 cases from northern Italy: a two-centre descriptive study. Lancet Infect Dis.

[4] Hanley B, Naresh KN, Roufosse C (2020). Histopathological findings and viral tropism in UK patients with severe fatal COVID-19: a post-mortem study. Lancet Microbe.

[5] Maiese A, Manetti AC, La Russa R, Di Paolo M, Turillazzi E, Frati P, Fineschi V (2021). Autopsy findings in COVID-19-related deaths: a literature review. Forensic Sci Med Pathol.

[6] Matschke J, Lütgehetmann M, Hagel C (2020). Neuropathology of patients with COVID-19 in Germany: a post-mortem case series. Lancet Neurol.

[7] Mokhtari T, Hassani F, Ghaffari N, Ebrahimi B, Yarahmadi A, Hassanzadeh G (2020). COVID-19 and multiorgan failure: a narrative review on potential mechanisms. J Mol Histol.

[8] Middeldorp S, Coppens M, van Haaps TF (2020). Incidence of venous thromboembolism in hospitalized patients with COVID-19. J Thromb Haemost.

[9] Quah P, Li A, Phua J (2020). Mortality rates of patients with COVID-19 in the intensive care unit: a systematic review of the emerging literature. Crit Care.

[10] Charlson ME, Pompei P, Ales KL MC (1987). A new method of classifying prognostic comorbidity in longitudinal studies: development and validation. J Chronic Dis.

[11] Kellum JA, Lameire N, Aspelin P (2012). Kidney disease: improving global outcomes (KDIGO) acute kidney injury work group. KDIGO clinical practice guideline for acute kidney injury. Kidney Int Suppl (2011).

[12] Zhou F, Yu T, Du R (2020). Clinical course and risk factors for mortality of adult inpatients with COVID-19 in Wuhan, China: a retrospective cohort study. Lancet.

[13] Wang J, Hajizadeh N, Moore EE (2020). Tissue plasminogen activator (tPA) treatment for COVID-19 associated acute respiratory distress syndrome (ARDS): a case series. J Thromb Haemost.

[14] Zhang Y, Xiao M, Zhang S (2020). Coagulopathy and antiphospholipid antibodies in patients with Covid-19. N Engl J Med.

[15] Panigada M, Bottino N, Tagliabue P (2020). Hypercoagulability of COVID-19 patients in intensive care unit: a report of thromboelastography findings and other parameters of hemostasis. J Thromb Haemost.

[16] Baicus C, Stoichitoiu LE, Pinte L, Badea C (2021). Anticoagulant protein S in COVID-19: the low activity level is probably secondary. Am J Ther.

[17] Mancini I, Baronciani L, Artoni A (2021). The ADAMTS13-von Willebrand factor axis in COVID-19 patients. J Thromb Haemost.

[18] Bazzan M, Montaruli B, Sciascia S, Cosseddu D, Norbiato C, Roccatello D (2020). Low ADAMTS 13 plasma levels are predictors of mortality in COVID-19 patients. Intern Emerg Med.

[19] Tiscia GL, Favuzzi G, De Laurenzo A (2020). Reduction of ADAMTS13 levels predicts mortality in SARS-CoV-2 patients. TH Open.

[20] Martinelli N, Montagnana M, Pizzolo F (2020). A relative ADAMTS13 deficiency supports the presence of a secondary microangiopathy in COVID 19. Thromb Res.

[21] Turner NA, Nolasco L, Ruggeri ZM, Moake JL (2009). Endothelial cell ADAMTS-13 and VWF: production, release, and VWF string cleavage. Blood.

[22] Cao WJ, Niiya M, Zheng XW, Shang DZ, Zheng XL (2008). Inflammatory cytokines inhibit ADAMTS13 synthesis in hepatic stellate cells and endothelial cells. J Thromb Haemost.

[23] Henry BM, Benoit SW, de Oliveira MH, Lippi G, Favaloro EJ, Benoit JL (2021). ADAMTS13 activity to von Willebrand factor antigen ratio predicts acute kidney injury in patients with COVID-19: Evidence of SARS-CoV-2 induced secondary thrombotic microangiopathy. Int J Lab Hematol.

[24] Scully M, Cataland SR, Peyvandi F (2019). Caplacizumab treatment for acquired thrombotic thrombocytopenic purpura. N Engl J Med.

[25] Callewaert F, Roodt J, Ulrichts H (2012). Evaluation of efficacy and safety of the anti-VWF Nanobody ALX-0681 in a preclinical baboon model of acquired thrombotic thrombocytopenic purpura. Blood.

[26] Peigne V, Azoulay E, Coquet I (2013). The prognostic value of ADAMTS13 (a disintegrin and metalloprotease with thrombospondin type 1 repeats, member 13) deficiency in septic shock patients involves interleukin-6 and is not dependent on disseminated intravascular coagulation. Crit Care.

[27] Wang Y, Chen J, Ling M, López JA, Chung DW, Fu X (2015). Hypochlorous acid generated by neutrophils inactivates ADAMTS13: an oxidative mechanism for regulating ADAMTS13 proteolytic activity during inflammation. J Biol Chem.

